# The relationship between Internet use and mental health of the elderly: Analysis of the differences between urban and rural

**DOI:** 10.1371/journal.pone.0280318

**Published:** 2023-01-26

**Authors:** Jinyan Xu, Qianqian Zhang

**Affiliations:** 1 College of Law and Public Management, Hunan University of Science and Technology, Xiangtan, Hunan, China; 2 College of Education, Hunan University of Science and Technology, Xiangtan, Hunan, China; Shanghai Jiao Tong University, CHINA

## Abstract

Internet use has an important impact on the elderly health. Based on the data of China General Social Survey (CGSS) in 2017, Model 4 and Model 14 in PROCESS were used to test the mechanism of Internet use on the mental health of the elderly, and further compare the differences between urban and rural elderly. The results are that Internet can positively predict the mental health of the whole sample and the urban elderly, but it has no significant predictive effect on the rural elderly; Internet can negatively predict the alienation of whole sample and urban and rural elderly; Alienation has a partly mediated effect between internet use and mental health of the whole elderly; "Internet using—alienation—mental health" the second path was moderated by embodied cultural capital in the whole sample and in the urban elderly. The conclusions are that Internet has a protective effect on the mental health of the elderly, and the mental health can be improved by reducing alienation. Increasing the use of the Internet and embodied cultural capital is an effective way to improve the mental health of the elderly. It is necessary to provide more internet access opportunities for the elderly, especially those in rural areas, increase the accessibility of embodied cultural capital, and bridge the digital divide between urban and rural elderly.

## Introduction

The period from 2020 to 2030 is a period of rapid development of aging. It is estimated that the elderly population in China will account for 23.57% of the total population by 2025 [1]. The aging of elderly groups is often accompanied by psychological problems such as anxiety, depression and loneliness. Due to the growth of age, the self stereotype of the elderly also increases, which leads to the negative self-development expectation of the elderly, and finally leads to the linear decline of mental health level [2]. With the acceleration of urbanization in China, the number of empty-nest elderly in rural areas has increased. Compared with cities, the mental health problems of rural elderly are more prominent, and the detection rate of depression symptoms is 1.76 times that of urban elderly [3]. Therefore, the mental health of the elderly has become a global social and public health issue.

With aging comes the rapid development of informatization. The use and popularization of the Internet has greatly enriched people’s life. The Internet has also become a tool for more and more elderly people to acquire news knowledge and communicate with their relatives and friends. The impact of Internet use on the mental health of the elderly has gradually received widespread attention from scholars. Scholars at home and abroad mainly have two views on the relationship between Internet use and mental health of the elderly. First, Internet use can promote mental health of the elderly. Shapira N et al. found that the mental health level of the elderly improved significantly after Internet training [4]. Some studies have also shown that the use of the Internet enhances the social connection of the elderly and reduces the sense of loneliness [5]. Another view is that Internet use will have an adverse impact on the mental health of the elderly, and that the elderly who use too much Internet have a higher sense of loneliness [6]. The inconsistency of research conclusions shows that the relationship between Internet use and mental health needs to be further explored in order to form a more convincing conclusion. At the same time, due to the inherent urban-rural dichotomy in China, non-Internet users are still mainly in rural areas, and the existence of the digital divide between urban and rural is likely to have an unequal impact on the mental health of the elderly in urban and rural areas, which is also an issue that needs to be further discussion.

At present, there is little literature in China that examines the mechanisms of the influence of Internet use on mental health, especially from the perspective of alienation and embodied cultural capital. With the growth of age, the elderly are gradually separated from the surrounding environment, resulting in a certain degree of alienation, which has a negative impact on their mental health and mental adjustment ability [7]. Throughout the existing literature, most studies have explored the relationship between Internet use and alienation using students as subjects, arguing that Internet use exacerbates students’ alienation and negatively affects their mental health [8]. With the popularity of the Internet, we should note that its numerous features increase the close ties and interactions between individuals and enhance subjective well-being while improving the quality of life. Based on this, can Internet use be an effective way to reduce alienation by bringing the elderly closer to society and family? Can the reduction of alienation also be a mechanism for the impact of Internet use on the mental health of the elderly? In addition, it has been argued in the literature that embodied cultural capital influences individuals’ choices of healthy lifestyles and can promote physical and mental health [9]. The essence of embodied cultural capital is the participation of social activities, which plays a certain role in promoting the healthy behavior of the elderly, maintaining intimate relations and reducing social alienation [10]. But what role does embodied cultural capital play in the mechanisms of influence on Internet use and mental health? Does the degree of negative impact on mental health triggered by alienation vary for the elderly with different levels of embodied cultural capital? Research on the relationship between Internet use and mental health among the elderly in China has mainly focused on older people as a whole, and comparative studies on the elderly in urban and rural areas are scarce. Based on the 2017 Chinese General Social Survey(CGSS) Data, the paper attempts to explore the direction and extent of the impact of Internet use by the elderly on their mental health, and analyze the mechanism of alienation and specific cultural capital in their relationship. On this basis, this paper analyzed the differences between urban and rural areas in the mechanism of Internet use affecting the mental health of the elderly according to compare urban and rural areas to provide targeted policy recommendations for improving the mental health level of the elderly.

## Literature review and theoretical framework

### Internet use and mental health of the elderly

Studies have shown that Internet use, as a new social skill, not only provides the elderly with opportunities to interact with others, but also enhances their mental health, and the Internet has become an important tool for re-socialization of the elderly. Internet use not only enables the elderly to conform to the demands of the times, but also allows them to have more close social connections through the Internet, which leads to higher life satisfaction and happiness [11]. Some studies have also found that Internet use has enabled the elderly to avoid emotional isolation and that those who use the Internet have lower rates of depression compared to those who do not use the Internet [12]. Research on rural elderly shows that Internet use can significantly increase the sense of security and access of the elderly, but this positive impact is lower than that of urban elderly [13]. Generally speaking, the elderly maintain close interaction with society through the use of the Internet, thus achieving re-socialization. In the process, the elderly not only gain a greater sense of security and alleviate feelings of helplessness, but also generate positive emotions, increase their sense of belonging and improve their quality of life.

Accordingly, this paper proposes hypothesis 1: Internet use will significantly enhance the mental health of the elderly(including urban and rural areas).

### Internet use and alienation

The term "alienation" emphasizes the individual’s subjective feelings of powerlessness, alienation and indifference. Most domestic scholars use the definition of alienation by Professor Yang Dong and others, that is, alienation is a negative emotion such as isolation, meaninglessness, and oppression due to the alienation of the individual from others and society in the social network [14]. As the elderly age, their physical functions decline and they are unable to meet with relatives and friends who are geographically distant, and due to accelerated globalization and urbanization, adult children may move far away from their aging parents to work in faraway places, making the elderly age alone. As a result, the alienation of the elderly has become a pressing concern. Fortunately, the emergence of Internet technology has provided a channel for older adults to learn new things and to learn about social progress, allowing them to have more experiences of close interaction with others and becoming a key to cracking the challenge of alienation among older adults in the context of population aging. a study by Sum et al. found that more frequent use of the Internet was associated with lower levels of social isolation [6]. By analyzing 6886 documents on Internet use, Khosravi et al. found that Internet use was effective in alleviating social isolation among the elderly [15], accessing the Internet not only helped to satisfy individuals’ social needs, but also enabled them to obtain online social support from it, enhancing their sense of belonging and thus reducing alienation. Thus, it is clear that Internet use, as a tool that can overcome spatial and temporal barriers, can enhance social interaction and reduce alienation among the elderly.

Accordingly, this paper proposes hypothesis 2: Internet use can significantly reduce the alienation of the elderly(including urban and rural areas).

### Alienation and mental health

Due to the decline of physical functions and cognitive abilities, the elderly gradually become disconnected from the outside world, breeding a sense of loneliness and leading to social isolation problems and a strong sense of alienation, thus undermining their mental health and quality of life. Especially in China’s rural areas, a large number of young and middle-aged laborers go to work in cities, empty nests and left-behind elderly people increase, and the problem of alienation becomes more prominent. In studies on alienation among rural left-behind children, poor college students, migrant children, ethnic minority secondary school students, and empty nesters, community and accompanying elderly, alienation was found to significantly reduce their well-being, and alienation among the elderly was a predictor of depression among the elderly, and the stronger the alienation, the more severe the depression and the lower the mental health level [16]. Therefore, the negative impact of alienation on mental health is obvious. Interpersonal indifference and the existence of a sense of distance from everything around is detrimental to the development of mental health in the elderly.

Accordingly, combined with the previous hypothesis analysis, this paper proposes hypothesis 3: Older adults’ alienation significantly decreases their mental health level, but Internet use contributes to mental health level by decreasing alienation, so alienation has a mediating effect between the relationship between Internet use and mental health, and for the urban and rural elderly, this mediating effect also exists.

### Moderating role of embodied cultural capital

The term "cultural capital" was first proposed by the French sociologist Pierre Bourdieu. He divided cultural capital into three dimensions: embodied cultural capital, Institutionalized cultural capital and objectified cultural capital. Embodied cultural capital is the information absorbed from the cultural environment and accumulated through practice or learning. For example, through reading newspapers, watching movies, doing some handicrafts, visiting museums and other cultural activities to accumulate embodied cultural capital. Institutionalized cultural capital is mainly reflected in the widely recognized symbols of the society, such as diplomas and vocational qualification certificates. Objectified cultural capital is mainly embodied in physical objects, such as books, records, paintings and so on [17]. Embodied cultural capital is acquired mainly through the participation of the elderly in cultural activities, whether it is the rural or urban elderly, activity participation has become one of the ways to promote their physical and mental health. Social activity theory suggests that the participation of the elderly in social activities can fully ensure the vitality and motivation of older people at the physical, psychological and social levels [18]. Compared with the rural elderly, the facilities for the urban elderly to participate in activities are more perfect and complete, which can produce greater health benefits. Research has shown that participation in activities not only provides older adults with emotional support, personal satisfaction and information needs, but also prevents social isolation and the negative health effects that can result from social isolation [19]. Individuals with higher cultural capital are more articulate and better able to communicate with others around them, and the reliable social network formed during the communication process can serve as a "buffer" and increase self-efficacy, thus contributing to mental health [20]. Moreover, cultural capital is a predictor of an individual’s healthy lifestyle. Individuals with higher cultural capital tend to participate in more physical exercise and recreational and cultural activities, such as watching exhibitions and listening to concerts, and the participation in these activities is more conducive to eliminating the negative effects of alienation on mental health. Therefore, for older adults, specific cultural capital is likely to reduce the negative effects of alienation on mental health.

Accordingly, this paper proposes hypothesis 4: Embodied cultural capital moderates the second half of the mediating process of "Internet using—alienation—mental health", and the negative effect of alienation on mental health decreases as the level of embodied cultural capital increases, for urban and rural elderly, this moderating effect also exists.

**Fig 1:**
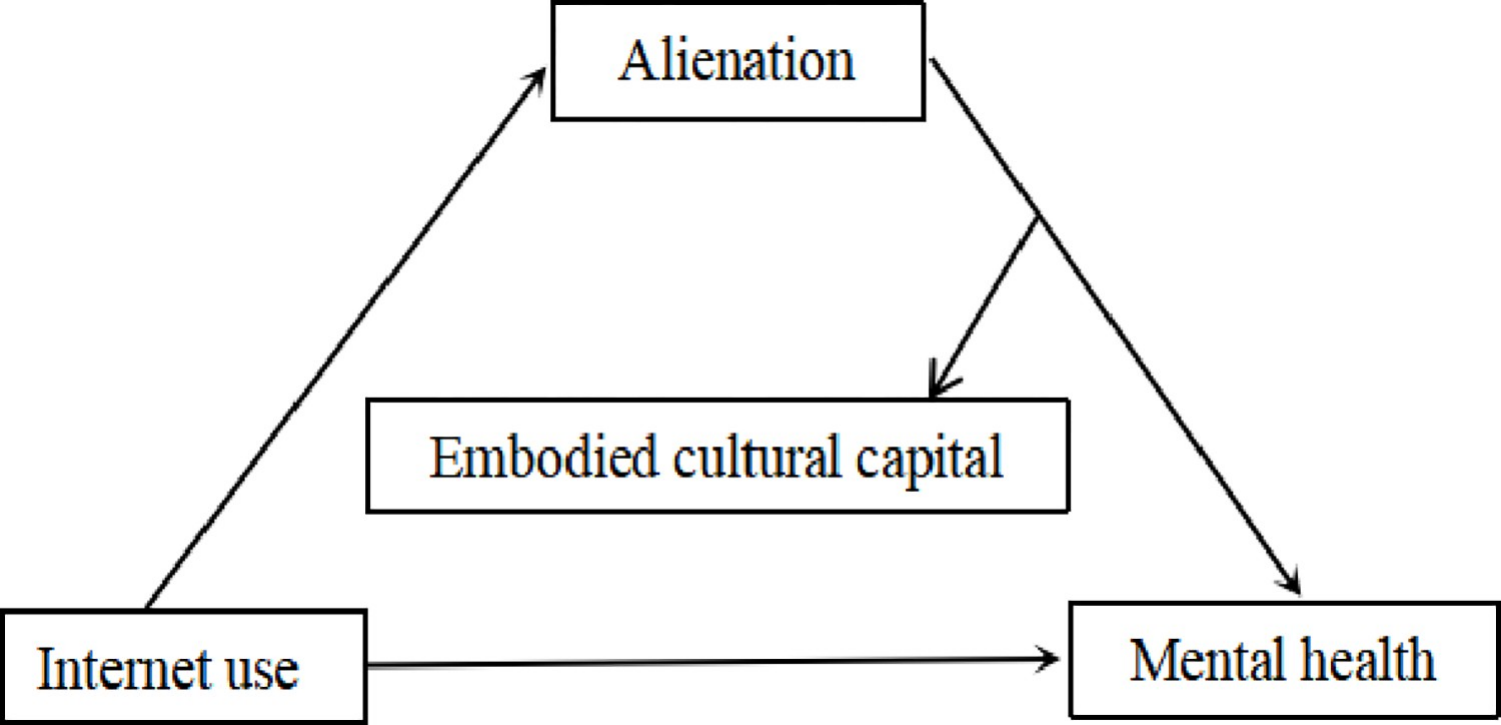
Hypothesis model. The mediation effect of alienation in the relationship between internet use and mental health. The moderation effect of embodied cultural capital in the relationship between alienation and mental health.

To sum up, this paper constructs a moderated mediation model to investigate the impact of Internet use on the mental health of the elderly and its mechanism, and analyzes the mediation effect of alienation in the relationship between them and the moderation effect of embodied cultural capital in the relationship between alienation and mental health. And the difference between urban and rural areas is analyzed. The theoretical hypothesis model is shown in Fig 1.

## Method

### Sources of date

The data comes from the 2017 Chinese general social survey data (CGSS), which is jointly implemented by the China Survey and data center of Renmin University of China and academic institutions all over the country. It is the earliest national, comprehensive and continuous academic survey project in China. The survey adopts the multistage sampling, and comprehensively collects the data of society, community, family and individual. The sample quality is high and the representativeness is strong. The research subjects include groups from 18 to 103 years old, covering 32 provinces (municipalities and autonomous regions), and the total number of samples for the survey subjects is 12,582. In this study, only the sample data of "60 years old and above" were selected. A total of 4372 elderly samples were obtained. Then, the samples with invalid answers (98 = unknown, 99 = refused to answer) or missing values in more than 70% of the items in the questionnaire were deleted. The percent of those invalid values is 4.77%. For the missing values, this study uses the mean to replace. This method is considered to be one of the most widely used interpolation methods so far, which can provide complete information for all cases and avoid selection errors. Finally, 4163 valid samples of elderly people were obtained.

### Variable manipulation

#### Dependent variable: Mental health of the elderly

Currently, the assessment of mental health of the elderly is mainly measured using multidimensional indicators and unidimensional indicators. The measurement of mental health level using unidimensional indicators is mainly based on designing a question to be answered by the survey respondents through self-assessment. Health self-assessment has been shown to be a valid measure of reflection of subjective health status [21], and the most commonly used measure of health status [22]. The data analysis for this study was based on the data of China General Social Survey (CGSS) in 2017, that measures the mental health of older adults using a unidimensional indicator approach. The question was designed as "In the past four weeks, how often did you feel depressed or frustrated?" The respondents were asked to answer the question with five options: "always, often, sometimes, rarely, and never", and were assigned a score of "1–5", with higher scores indicating higher mental health. In most of the existing studies, several scholars have also used this method to measure the mental health level of older adults [23, 24], and have come up with convincing conclusions. Therefore, in this study, a unidimensional self-rated mental health status was also used to measure the mental health level of the elderly in conjunction with the questionnaire survey.

#### Independent variable: Internet use

The independent variable of this paper is Internet use. The survey question of Internet use frequency in CGSS questionnaire is "what is the use of Internet (including mobile Internet) in the past year?", The answer is "never, rarely, sometimes, often, very frequently", and the value is "1–5". The higher the score, the higher the frequency of Internet use.

#### Mediating variable: Alienation

According to Yang et al, alienation has two levels of meaning; the first level of meaning refers to the direct emotional experience resulting from the alienation of the subject from the object, such as the alienation from relatives and friends; the second level of meaning is the complexified alienation, which is the alienation between the individual and human warmth, caring help, and the self, such as the self-perception of being isolated and left out [14]. Currently, Heping Xu’s measurement of detachment is more representative. He measured alienation from two dimensions of object-alienation and self-alienation, as well as seven indicators such as the frequency of contact with relatives and friends, and used factor analysis to measure the score of alienation [25]. Based on Yang Dong’s definition of the two levels of alienation, and drawing on Xu Haiping’s method for measuring alienation, this paper measures the alienation of older adults by selecting seven questions from the 2017 CGSS questionnaire that best represent these two levels (see Table 1). The results of factor analysis showed that the *KMO* of 7 items was 0.69>0.50, and the Bartlett’s test of sphericity *p*<0.001, indicating that factor analysis was suitable. The cumulative variance interpretation rate of the two factors (object-alienation and self-alienation) was 58.43%. The items of alienation measurement in this study were selected based on the relevant items of the questionnaire survey. The three items of self-alienation factor were measured by scale questions. The value of Cronbach’s alpha coefficient is 0.88. But the 4 items of object-alienation factor do not belong to the scale measurement, so it is not suitable for the reliability coefficient test. The data source of this study is a large-scale nationwide survey organized by Renmin University of China. The survey objects are representative. Moreover, the questionnaire was designed by professional authoritative scholars, and the survey process was conducted under scientific and standardized conditions. The investigators also received strict professional training which can ensure the reliability and stability of the questionnaire data. The alienation score is the sum of 7 items, with a total score of 7–47. The higher the score, the higher the degree of alienation. 7–20 is low level, 21–33 is medium level, and 34–47 is high level.

**Table 1:** Operational definition of alienation. The alienation contains two levels: object-alienation and self-alienation and a total of 7 questions remained.

Mediating variable	Alienation levels	Questionnaire item	Ranging
Alienation	Object-alienation	Think about the family member (excluding siblings) who has the most contact with you, how often do you contact him/her?	Everyday = 1 Several times a week = 2 Once a week = 3 2 or 3 times a month = 4 once a month = 5 several times a year = 6 rarely = 7 never = 8
		Think about your closest friend you contact most, how often do you contact him / her?	
		Think about your siblings you contact most, how often do you contact him / her?	
		Thinking about your most contacted adult child, how often do you have contact with him/her?	
	Self-alienation	How often have you felt left out in the past four weeks?	Never = 1 Rarely = 2 Sometimes = 3 Often = 4 Very frequently = 5
		How often have you felt isolated from others in the past four weeks?	
		How often have you felt unaccompanied in the past four weeks?	

Note: For the elderly without siblings/adult children/close friends/family, their contact frequency is classified as "never".

#### Moderating variable: Embodied cultural capital

Bourdie’s cultural capital theory states that the acquisition of specific cultural capital is premised on internalization, a process that requires time investment as well as individual personal effort, and is a spiritual acquisition that is reflected in enduring dispositions and physical and mental qualities, including values, preferences, behavioral norms, and operational skills. When Wang Hongbo, a domestic scholar, measured the embodied cultural capital of rural residents, he selected eight cultural activities, such as watching TV or DVDs, watching movies, reading books/newspapers/magazines, watching performances and exhibitions, listening to concerts, participating in physical exercise, watching sports competitions live, doing handicrafts (embroidery, woodworking) [26]. This study combines Buedi’s cultural capital theory and Wang Hongbo’s measurement of embodied cultural capital. Eight activities were selected from the CGSS questionnaire to measure the embodied cultural capital of the elderly: watching TV, going out to the movies, reading books/newspapers/magazines, going to performances and exhibitions, listening to music at home, physical exercise, watching sports games live, and doing crafts. These activities were gradually accumulated mainly through individual cultural attitudes and social practices, and the corresponding questionnaire question was "In the past year, did you often participate in the following activities in your free time?" (participated = 1, did not participate = 0), and the scores of the eight activities were summed up to the embodied cultural capital score of the elderly, which ranged from 0 to 8, and the higher the score, the higher the level of embodied cultural capital of the elderly.

#### Control variables: Social demographic characteristics

Most studies have shown that gender, age, education, residence, and physical health status among individual background characteristics variables affect the frequency of Internet use and mental health of older adults [27], in addition to their economic level, marital status, and whether they live with their children are also important factors that affect their mental health [28]. Therefore, the control variables selected for individual characteristics in this study mainly included age, that is, CGSS survey year minus respondent’s year of birth(60–69 = 1,70–79 = 2,≥80 = 3), gender (female = 0, male = 1), education (primary school and below = 1, junior middle school = 2, senior high school = 3, college = 4, undergraduate and above = 5) and residence (rural = 0, urban = 1), marital status (no spouse = 0, with spouse = 1), economic level (far below average = 1, below average = 2, average = 3, far above average = 4, above average = 5), residence style (not living with children = 0, living with children = 1), and physical health (very unhealthy = 1, relatively unhealthy = 2, average = 3, relatively healthy = 4, very healthy = 5).

#### Analysis process

Firstly, descriptive statistics of sample variables; Secondly, Pearson correlation analysis was used to test the relationship between variables; Finally, based on the test method of mediation and moderation effect in the process macro program written by Hayes [29], Model 4 and Model 14 are used to test the mechanism of Internet use on the mental health of the elderly. Model 4 examines the relationship between Internet use, alienation, and mental health and the mediating effect of alienation. Model 14 tests the moderation effect of embodied cultural capital. Bias-corrected Bootstrap is used to test the significance of the regression coefficient. The repeated sampling is set for 5000 times, and the confidence interval is 95%. When the confidence interval does not contain 0, the result is significant. Statistical analyses were conducted with IBM SPSS statistics 26.

## Results

### Common method biases test

The 2017 CGSS questionnaire mainly uses self-report to collect data, which may lead to common method biases. In this study, Harman single factor test was used to count the biases of the common method. The data show that there are 6 factors with eigenvalues greater than 1, the explained variation of the first factor is 13.71% (< 40%), indicating that there is no serious common method biases in the survey data.

### Descriptive statistics of variables

Descriptive statistical results of sample characteristics are shown in Table 2. Among 4,163 elderly people, 59.1% are urban elderly and 40.9% are rural elderly. Women (51.2%) are more than men (48.8%); The elderly aged 60–69 are the most, accounting for 58.7%; More than half of the elderly have primary school education or below (56.4%); Most of the elderly with spouses, accounting for 73.6%; The economic situation is at a medium level (2.48±0.79); Only 3.6% of the elderly live with their children; The physical health score of the elderly is 2.98±1.08, which is in the middle level; Self-rated mental health was 3.73±1.03, which was above the average level. 76.8% of the elderly said that they never used the Internet, with an average of 1.62 points and a large variance of 1.55, indicating that the frequency of Internet use by the elderly is low, and there are great individual differences in their usage; The score of alienation of the elderly is 22.93±3.81, which is in the middle level; The overall score of the embodied cultural capital of the elderly is 2.89±1.91, which is in the lower middle level. In addition, the mental health level, Internet usage frequency and embodied cultural capital level of rural elderly are significantly lower than those of urban elderly(*p*<0.001). Compared with the urban elderly, the rural elderly have a higher degree of alienation (*p*<0.001).

**Table 2:** Descriptive statistics of sample variables.

Variable	Full sample (N = 4163)	Rural (n = 1703)	Urban (n = 2460)	t / χ^2^
Gender(n(%))				12.10**
female	2132(51.2)	817(48.0)	1315(53.5)	
male	2031(48.8)	886(52.0)	1145(46.5)	
Age(n(%))				27.16***
60–69	2445(58.7)	1038(61.0)	1407(57.2)	
70–79	1223(29.4)	516(31.3)	707(28.7)	
≥80	495(11.9)	149(8.7)	346(14.1)	
Marital status(n(%))				0.91
no spouse	1101(26.4)	437(25.7)	664(27.0)	
with spouse	3062(73.6)	1266(74.3)	1796(73.0)	
Residence style(n(%))				10.34**
not living with children	4014(96.4)	1661(97.5)	2353(95.7)	
living with children	149(3.6)	42(2.5)	107(4.3)	
Education(n(%))				
primary school and below	2348(56.4)	1324(77.7)	1024(41.6)	605.23***
junior middle school	1029(24.7)	297(17.4)	732(29.8)	
senior high school	504(12.1)	72(4.2)	432(17.6)	
college	163(3.9)	7(0.4)	156(6.3)	
undergraduate and above	119(2.9)	3(0.2)	116(4.7)	
Economic level	2.48±0.79	2.28±0.81	2.62±0.76	-13.53***
Physical health	2.98±1.08	2.75±1.09	3.14±1.03	-11.82***
Mental health	3.73±1.03	3.48±0.99	3.91±1.01	-13.72***
Internet use	1.62±1.25	1.14±0.61	1.95±1.45	-21.89***
Alienation	22.93±3.81	23.53±3.88	22.51±3.70	8.55***
Embodied cultural capital	2.89±1.91	2.02±1.48	3.49±1.94	-26.33***

Significance levels

*p<0.05

**p<0.01

***p<0.001.

#### Correlation analysis among variables

Pearson correlation analysis was used to test the relationship among Internet use, mental health, alienation and embodied cultural capital. The results of correlation analysis in Table 3. Internet use has a significant positive correlation with embodied cultural capital and mental health, and a significant negative correlation with alienation; Alienation has a significant negative correlation with embodied cultural capital and mental health; Embodied cultural capital is positively correlated with mental health.

**Table 3:** Correlation coefficient matrix of main variables.

Variables	1	2	3	4	5	6	7	8	9	10	11	12
1.Gender	1											
2.Age	-0.10	1										
3.Residence	-0.05**	0.06***	1									
4.Education	0.14***	-0.06***	0.37***	1								
5.Marital status	0.16***	-0.31***	-0.02	0.11***	1							
6.Economic level	0.02	0.03	0.21***	0.27***	0.08***	1						
7.Residence style	-0.04*	-0.07***	0.05**	0.06***	-0.01	0.02	1					
8.Physical health	0.10***	-0.07***	0.18***	0.20***	0.06***	0.25***	0.04*	1				
9.Internet use	0.07***	-0.18***	0.32***	0.49***	0.11***	0.19***	0.05**	0.19***	1			
10.Alienation	-0.01	0.06***	-0.12***	-0.16***	-0.15***	-0.16***	0.01	-0.15***	-0.15***	1		
Embodied cultural capital	0.02	-0.10***	0.38***	0.46***	0.11***	0.28***	0.05**	0.24***	0.45***	-0.17***	1	
12.mental health	0.09***	0.01	0.21***	0.22***	0.07***	0.26***	0.01	0.44***	0.18***	-0.20***	0.22***	1

Significance levels

*p<0.05

**p<0.01

***p<0.001.

#### A model of the relationship between Internet use, alienation, and mental health of older adults

According to Wen Zhonglin and Ye Baojuan [30], PROCESS Model 4 was used to establish three regression equations for testing the mediation mechanism. Equation 1 estimated the direct effect of Internet use on mental health. Equation 2 estimated the predictive effect of Internet use on alienation. And equation 3 estimated the mediating effect of alienation between Internet use and mental health. Tables 4–6 were the mediating test results of the whole sample, urban and rural elderly respectively. The independent variable was Internet use, the dependent variable was mental health, and the mediating variable was alienation, with controls for individual characteristic variables such as gender and age. The dependent variable in equation 1 and 3 was mental health, and the dependent variable in equation 2 was alienation. Both equations 1 and 2 put in only Internet use and control variables, and equation 3 put in both Internet use, alienation, and control variables (see Tables 4–6).

**Table 4:** Regression results of the relationship among Internet use, alienation and mental health of the elderly (Full sample). All variables entered the regression equation after being standardized.

Variable	Equation 1 (Y: Mental health)		Equation 2 (M: Alienation)		Equation 3 (Y: Mental health)	
	*β*	95%*CI*	*β*	95%*CI*	*β*	95%*CI*
Internet use	0.04*	[0.01,0.07]	-0.04**	[-0.07,-0.02]	0.03**	[0.00,0.06]
Alienation					-0.14***	[-0.17,-0.09]
Gender	0.07**	[0.01,0.12]	0.04	[-0.00,0.08]	0.07**	[0.02,0.13]
Age	0.01***	[0.00,0.01]	0.01	[-0.00,0.01]	0.01***	[0.00,0.01]
Residence	0.18***	[0.11,0.23]	-0.07**	[-0.12,-0.02]	0.16***	[0.10,0.22]
Education	0.06**	[0.02,0.09]	-0.05***	[-0.07.-0.02]	0.05**	[0.02,0.08]
Marital status	0.10**	[0.04,0.16]	-0.20***	[-0.25,-0.15]	0.07*	[0.01,0.14]
Economic level	0.15***	[0.11,0.18]	-0.08***	[-0.11,-0.06]	0.14***	[0.09,0.17]
Residence style	-0.11	[-0.25,0.03]	0.12*	[0.01.0.24]	-0.09	[-0.24,0.05]
Physical health	0.35***	[0.32,0.37]	-0.06***	[-0.08,-0.04]	0.34***	[0.31,0.36]
*R* ^*2*^	0.24		0.07		0.25	
*F*	142.27***		36.77***		134.52***	

Significance levels

*p<0.05

**p<0.01

***p<0.001. *N* = 4163.

**Table 5:** Regression results of the relationship among Internet use, alienation and mental health of urban elderly. All variables entered the regression equation after being standardized.

Variable	Equation 1 (Y: Mental health)		Equation 2 (M: Alienation)		Equation 3 (Y: Mental health)	
	*β*	95%*CI*	*β*	95%*CI*	*β*	95%*CI*
Internet use	0.05**	[0.01,0.08]	-0.08***	[-0.11,-0.04]	0.05**	[0.01,0.08]
Alienation					-0.04	[-0.07,0.00]
Gender	0.07	[-0.00,0.14]	0.12**	[0.04,0.19]	0.07	[-0.00,0.14]
Age	0.06*	[0.01,0.11]	0.05	[-0.01,0.11]	0.06*	[0.01,0.11]
Education	0.05*	[0.01,0.08]	-0.05**	[-0.09,-0.01]	0.04*	[0.01,0.08]
Marital status	-0.09*	[-0.17,-0.00]	0.00	[-0.08,0.09]	-0.09*	[-0.17,-0.00]
Economic level	0.15***	[0.10,0.20]	-0.13***	[-0.18,-0.07]	0.14***	[0.09,0.19]
Residence style	-0.11	[-0.29.0.06]	0.22*	[0.04,0.41]	-0.11	[-0.29.0.06]
Physical health	0.33***	[0.29,0.37]	-0.07***	[-0.11,-0.04]	0.33***	[0.29,0.36]
*R* ^*2*^	0.18		0.05		0.17	
*F*	65.42***		15.89***		58.58***	

Significance levels

*p<0.05

**p<0.01

***p<0.001. *N* = 2460.

**Table 6:** Regression results of the relationship among Internet use, alienation and mental health of rural elderly. All variables entered the regression equation after being standardized.

Variable	Equation 1 (Y: Mental health)		Equation 2(M: Alienation)		Equation 3 (Y: Mental health)	
	*β*	95%*CI*	*β*	95%*CI*	*β*	95%*CI*
Internet use	-0.04	[-0.13,0.05]	-0.12*	[-0.22,-0.01]	-0.05	[-0.13,0.04]
Alienation					-0.07**	[-0.11,-0.03]
Gender	0.07	[-0.01,0.15]	-0.00	[-0.10,0.09]	0.07	[-0.01,0.15]
Age	0.10**	[0.04,0.17]	0.04	[-0.04,0.11]	0.10**	[0.04,0.17]
Education	0.11***	[0.04,0.19]	-0.11*	[-0.19,-0.02]	0.11***	[0.03,0.18]
Marital status	-0.03	[-0.13,0.07]	0.11	[-0.01,0.22]	-0.02	[-0.11,0.07]
Economic level	0.14***	[0.09,0.20]	-0.13***	[0.20,-0.06]	0.13***	[0.08,0.18]
Residence style	-0.10	[-0.36,0.16]	-0.16	[-0.47,0.15]	-0.11	[-0.37,0.14]
Physical health	0.36***	[0.32,0.39]	-0.07**	[-0.11,-0.02]	0.36***	[0.32,0.39]
*R* ^*2*^	0.23		0.03		0.24	
*F*	63.32***		7.40***		57.83***	

Significance levels

*p<0.05

**p<0.01

***p<0.001. *N* = 1703.

Table 4 results showed that Internet use significantly predicted mental health positively (*β* = 0.04, *p*<0.05) and significant negative prediction of alienation (*β* = -0.04, p<0.01), so the first half of hypothesis 1 and hypothesis 2 were passed; when Internet use and alienation were entered into the regression equation at the same time, the positive predictive effect of Internet use on mental health was still significant (*β* = 0.03, *p*<0.05), and alienation significantly negatively predicted mental health (*β* = -0.14, *p*<0.001), and the results of Bias-corrected Bootstrap showed that the mediating effect of alienation in Internet and mental health was significant, with 95% confidence interval [0.002, 0.010], and the mediating effect was 0.006, and the mediation effect accounts for 15.38% of the total effect (0.039), so alienation has a partially mediating effect between Internet use and mental health of the elderly, so the first half of hypothesis 3 test was passed. In addition, among the individual control variables, gender, age, residence, education, marital status, economic level, and physical health were all significant positive predictors of mental health among older adults.

The results in Table 5 show that Internet use of urban elderly can significantly positively predict mental health (*β* = 0.05, *p*<0.01) and negatively predict alienation (*β* = -0.08, *p*<0.001). When Internet use and alienation enter the regression equation at the same time, the positive predictive effect of Internet use on mental health is still significant (*β* = 0.05, *p*<0.01), but the predictive effect of alienation on mental health is not significant (*β* = -0.04, *p*>0.05). Therefore, alienation does not play a mediated role between internet use and mental health of urban elderly. The second half of hypothesis 3 is not passed.

The results in Table 6 show that Internet use has no significant impact on the mental health of rural elderly (*β* = -0.04, *p*>0.05), but Internet use in rural elderly significantly negatively predicts their alienation (*β* = -0.12, *p*<0.05). Moreover, when Internet use and alienation enter the regression equation at the same time, Internet use has no significant predictive effect on mental health (*β* = -0.05, *p*>0.05), while alienation significantly negatively predicts mental health (*β* = -0.07, *p*<0.01), so alienation does not play a mediated role between Internet use and mental health of rural elderly. Internet use has a positive predictive effect on the mental health of the rural elderly in hypothesis 1, and the second half of hypothesis 3 are not passed.

#### Moderated mediation model test

The PROCESS Model14 was used to test the moderated mediation effect. To avoid multicollinearity, all predictor variables were standardized, individual characteristics variables were controlled for, and two regression equations were established (see Tables 7 and 8). Equation 1 estimated the predictive effect of the Internet on alienation, and Equation 2 estimated the moderating effect of specific cultural capital on Internet use, alienation and mental health. Tables 7 and 8 show the results of moderated mediation model test of the whole sample and the urban and rural elderly respectively. In equation 1, the dependent variable was alienation, and Internet use and control variables were put in. In equation 2, the dependent variable was mental health, after putting in the Internet use, alienation, embodied cultural capital, and interaction terms of alienation with embodied cultural capital, and controlling for older adults’ personal characteristics variables. The results in Table 7 show that Internet use significantly and negatively predicting alienation (*β* = -0.041, *p*<0.01), the product term of alienation with embodied cultural capital was a significant predictor of mental health (*β* = 0.041, *p*<0.05), the moderating effect of embodied cultural capital is significant.

**Table 7:** Moderated mediation test of Internet use on mental healthFull sample).

Variables	Equation 1 (Y: Alienation)		Equation 2 (Y: Mental health)	
	*β*	95%*CI*	*β*	95%*CI*
Gender	0.041	[-0.002,0.084]	0.075**	[0.021,0.130]
Age	0.001	[-0.002,0.004]	0.007***	[0.004,0.012]
Residence	-0.067**	[-0.115,-0.020]	0.147***	[0.086,0.209]
Education	-0.049***	[-0.074,-0.023]	0.044**	[0.012,0.077]
Marital status	-0.201***	[-0.251,-0.149]	0.069*	[0.005,0.135]
Economic level	-0.084***	[-0.113,-0.056]	0.131***	[0.094,0.167]
Residence style	0.124*	[0.009,2.238]	-0.105	[-0.248,0.038]
Physical health	-0.062***	[-0.083,-0.041]	0.033***	[0.307,0.359]
Internet use	-0.041**	[-0.066,-0.015]	0.028	[-0.004,0.061]
Alienation			-0.133**	[-0.171,-0.094]
Embodied cultural capital(ECC)			0.032*	[0.001,0.065]
Alienation×ECC			0.041*	[0.005,0.077]
*R* ^*2*^	0.074			0.246
*F*	36.768***			103.013***

Significance levels

*p<0.05

**p< 0.01

***p<0.001. *N* = 4163.

**Table 8:** Moderated mediation effect of Internet use on mental health of urban and rural elderly.

Variables	Rural (N = 1703)				Urban (N = 2760)			
	Equation 1 (M: Alienation)		Equation 2 (Y: Mental health)		Equation 1 (M: Alienation)		Equation 2 (Y: Mental health)	
	*β*	95%*CI*	*β*	95%*CI*	*β*	95%*CI*	*β*	95%*CI*
Gender	-0.01	[-0.10,0.09]	0.07	[-0.01,0.15]	0.12**	[0.04,0.19]	0.07*	[0.00,0.14]
Age	0.04	[-0.04,0.11]	0.10**	[0.04,0.17]	0.05	[-0.01,0.11]	0.07*	[0.01,0.11]
Education	-0.11*	[-0.19,-0.02]	0.10*	[0.02,0.17]	-0.05**	[-0.09,-0.01]	0.03	[-0.00,0.07]
Marital status	0.11	[-0.01,0.22]	-0.02	[-0.12,0.08]	0.00	[-0.08,0.09]	-0.09*	[-0.17,-0.00]
Economic level	-0.13***	[0.20,-0.06]	0.13***	[0.08,0.18]	-0.13***	[-0.18,-0.07]	0.14***	[0.09,0.19]
Residence style	-0.16	[-0.47,0.15]	-0.11	[-0.37,0.15]	0.22*	[0.04,0.41]	-0.12	[-0.29,0.05]
Physical health	-0.07**	[-0.11,-0.02]	0.35***	[0.32,0.39]	-0.07***	[-0.11,-0.04]	0.32***	[0.29,0.36]
Internet use	-0.12*	[-0.22,-0.01]	-0.05	[-0.14,0.04]	-0.08***	[-0.11,-0.04]	0.04*	[0.00,0.07]
Alienation			-0.05*	[-0.10,-0.01]			-0.05*	[-0.09,-0.01]
Embodied cultural capital(ECC)			-0.02	[-0.04,0.07]			0.04*	[0.00,0.08]
Alienation×ECC			0.02	[-0.03,0.07]			0.04*	[0.01,0.08]
*R* ^*2*^	0.03		0.24		0.05		0.18	
*F*	7.40***		47.42***		15.89***		48.94***	

Significance levels

*p<0.05

**p< 0.01

***p<0.001.

**Fig 2:**
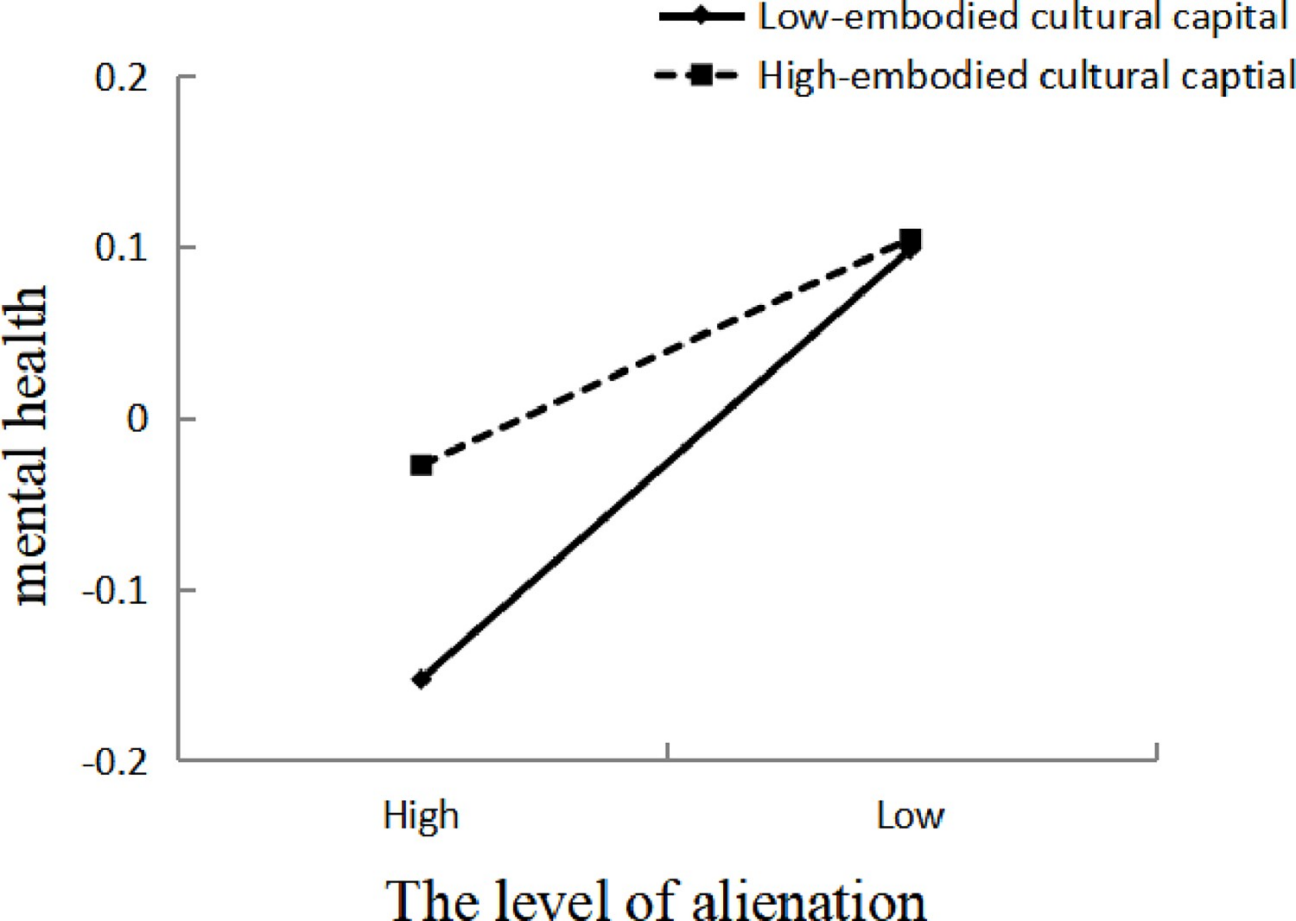
The moderation effect of embodied cultural capital. The level of alienation is divided into high and low, with high representing *M*+*SD*, i.e., above the mean, and low representing *M*-*SD*, i.e., below the mean, and since the variables are standardized, the mean value is 0, i.e., high and low are ±1.

In order to further explain the moderation effect of embodied cultural capital, the embodied cultural capital is divided into high and low groups according to its average plus or minus a standard deviation (*M*±*SD*), and a simple slope test is performed (Fig 2). The results showed that there was a significant negative predictive effect of alienation on mental health when the level of embodied cultural capital was low (*B*_simple_ = -0.174, *t* = -6.683, *p*<0.001, *95% CI* [-0.225, -0.123]), And when the level of embodied cultural capital is high, the negative predictive effect of Internet use on alienation is mitigated (*B*_simple_ = -0.092, *t* = -3.317, *p*<0.001, *95% CI* [-0.146, 0.038]); that is, negative effects on mental health triggered by alienation diminish as the level of embodied cultural capital increases, and the first half of hypothesis 4 test was passed.

The results in Table 8 show that for the rural elderly, Internet use significantly negatively predicts alienation (*β* = -0.12, *p*<0.05), and the product of alienation and embodied cultural capital has no significant predictive effect on mental health (*β* = 0.02, *p*>0.05), and embodied cultural capital has no moderating effect. That embodied cultural capital has a moderating effect on the Internet use and mental health of rural elderly in hypothesis 4 is not passed. For the urban elderly, the Internet use significantly negatively predicts alienation (*β* = -0.08, *p*<0.001), alienation significantly negatively predicts mental health (*β* = -0.05, *p*<0.05), and the product of alienation and embodied cultural capital significantly predicts mental health (*β* = 0.04, *p*<0.05), Therefore, the embodied cultural capital plays a significant moderated role.

Using the same method, making a simple slope test on the moderated effect of the embodied cultural capital of the urban elderly, and the result is consistent with the moderated effect of the full sample. The hypothesis 4 that embodied cultural capital has a moderated effect on the relationship between Internet use and mental health of the urban elderly has been verified.

## Conclusion and discussion

Based on the 2017 CGSS survey data, this paper attempts to explore in depth, for the first time, the influence mechanism of the Internet on mental health of the elderly from the perspective of alienation and embodied cultural capital, while analyzing the moderating role of embodied cultural capital in the influence mechanism. Furthermore, it makes a comparative analysis of its mechanism between urban and rural areas. The results show that, for the whole elderly, Internet use can directly improve their mental health level, and alienation is a risk factor for their mental health. Internet use affects mental health through the mediating role of alienation, and the second half of this mediating process is moderated by embodied cultural capital; The negative effect of alienation on mental health was greater when the level of embodied cultural capital was low, and the effect of alienation on mental health slowed down as the level of embodied cultural capital increased. The influence mechanism of Internet use on the mental health of the elderly is partially different between urban and rural areas. The research conclusion provides theoretical and practical reference for relevant administrative departments to formulate differentiated strategies for promoting the mental health of the elderly in urban and rural areas.

Firstly, the direct impact of Internet use on the mental health of the elderly is different between urban and rural areas. For the whole sample and urban elderly, Internet use has a positive impact on the mental health of the elderly, which is consistent with the findings of Zhao et al. [31]. However, in rural areas, Internet use does not have a direct effect on the mental health of the elderly. This may be due to the fact that compared to urban areas, rural elderly live in remote mountain villages in particular, where Internet coverage is low and network infrastructure is more backward, and older adults lack the hardware conditions to use the Internet. At the same time, rural elderly people have a low level of education, most of them have elementary school education or below, and have been living in a relatively single and closed environment for a long time, and their ideology is also more conservative and unwilling to accept and learn new things and new knowledge. Even in areas where the Internet is available, the skills and willingness of the elderly to use various network tools such as smartphones are still very low. In addition, most elderly people have declining labor ability, lack stable income sources, and lack the financial conditions to purchase smartphones for use. Therefore, in rural areas, Internet use does not play a positive role in the mental health of older adults as it should. The Internet is one of the protective factors for the mental health of older adults, and the inequality in the mental health of older adults in urban and rural areas incorporates factors of the urban-rural digital divide. Therefore, relevant authorities should enhance the popularity and age-appropriateness of mobile Internet and public service websites, especially the popularity and training of Internet usage knowledge for older female groups in rural areas, without spouses and with lower education levels. To further promote digital literacy education for rural elderly people, social workers and children play the role of leading the Internet life of the elderly, and should encourage more elderly people to use the Internet and experience the fun of Internet life to enhance their willingness to join the Internet. In addition, some smart network devices can be modified to make them more suitable for the elderly.

Secondly, alienation has a negative impact on the mental health of the elderly in both urban and rural areas. looking-glass self theory believes that the evaluation and attitude of others towards oneself is a "mirror" reflecting the self, through which individuals know and grasp themselves, forming the "self in the mirror", and this feeling of loneliness and isolation undoubtedly lays the foundation for the elderly to form a negative view of self [32], which leads to internalized psychological problems. The elderly with a higher sense of alienation are generally aging, disabled, empty nesters and have fewer children, this situation is particularly prominent in rural areas, which not only breeds a sense of loneliness, but also contributes to the generation of stereotypes (illness, retardation, loneliness, worthlessness, etc.) that lead to negative emotional experiences. As a result, the elderly are more susceptible to poor mental health outcomes due to alienation. Therefore, it is imperative to mitigate alienation in older adults. Society should focus on those older adults with higher levels of alienation, such as those in rural areas, those without spouses, those living alone, those with lower levels of education and economic status, and those with poorer physical health, and provide more care, comfort, and social support to these special groups of the elderly.

Thirdly, Internet use has significantly reduced the sense of alienation of the elderly in urban and rural areas. Socioemotional selectivity theory suggests that as individuals age, their time to perceive the future becomes increasingly limited and their social goals shift from acquisition knowledge, which aims to gain knowledge, to regulation of emotions, which aims to focus on the meaning and intimacy of life [33]. The elderly anticipate limited time and will try to consolidate interpersonal relationships for emotional support. The Internet, as a new way of life, ensures the smooth implementation of the emotional interaction goals of the elderly, makes the interpersonal network of the elderly closer, improves the recognition of family relationships, and also satisfies the interaction needs and affection needs of the elderly, enhancing the emotional connection with family members as well as friends, thus avoiding social isolation and reducing their sense of alienation. Therefore, it is necessary to strengthen the popularity of the Internet among the elderly, and to balance the gap between urban and rural areas; at the same time, some brochures should be distributed in the community to guide the elderly to use smart devices and help them cross the "digital divide".

Fourthly, alienation has a partially mediated effect between Internet use and mental health in the whole sample, but the mediated effect is not significant in urban and rural elderly. Individual aging is often accompanied by a contraction of real-life social circles, which can significantly increase alienation in older adults [34]. The Internet, as an important social media, can break the limitation of the activity range of the elderly group and expand their social channels and social scope, which can effectively improve the social frequency and increase the opportunities for the elderly to learn knowledge, leisure and entertainment and home aging, so that the elderly can fully enjoy the benefits brought by the Internet to their senior life. On the contrary, those older people who have not used the Internet, along with the gradual deterioration of their social functions, will lead to a decrease in their level of social participation and social interaction, and their level of alienation is higher compared to those who use the Internet, which is ultimately detrimental to the development of their mental health.

In the case of both rural and urban older adults, alienation does not play a mediating role between Internet use and mental health. This may be due to the fact that in rural areas, as mentioned earlier, rural older adults lack the conditions and motivation to use the Internet, which naturally does not play a role in mitigating alienation as it should. In urban areas, where older adults live, a variety of amenities are more well-equipped, recreational and social interaction options are increased, and abundant activity resources all play a role in alleviating alienation, possibly even more than Internet use. In addition to Internet use, other factors that contribute to the alleviation and elimination of alienation need to be further explored. Therefore, in order to have a better Internet experience for the elderly and enrich their lives, some applications suitable for the elderly can be launched, and some contents of interest to the elderly such as square dance instruction and opera can be set up to promote the frequency of using the Internet for the elderly, thus reducing their alienation level and promoting positive development of mental health.

Fifthly, in the path of “Internet use—alienation—mental health”, the second half path was moderated by embodied cultural capital. But it has no significant moderated effect in rural areas. This suggests that embodied cultural capital can significantly reduce the negative effects of alienation on mental health. The results support the risk buffer model, according to which the negative impact of risky factors can be buffered by increasing an individual’s favorable resources [35]. Older people with a high level of embodied cultural capital are able to actively participate in social and cultural activities. Such group activities can alleviate the isolation of older people and reduce the risky effect of alienation on mental health. For rural older adults, the moderating effect of specific cultural capital is not significant. This may be due to the fact that with the accelerated urbanization process in China, more and more rural elderly people are left behind and their social life is more closed, which hinders their social participation process [36]. Most of the rural elderly have gone through hard and difficult years, and they are more concerned about how to survive than how to enjoy life. Therefore, in their later years, even if local cultural and recreational facilities are provided, their awareness of participating in using them is still very weak, and specific cultural capital does not play a corresponding role in mental health. The relevant social departments and staff should focus on the accessibility of embodied cultural capital for older people, such as holding regular film screenings, building cultural exhibition halls and gymnasiums suitable for older groups, and actively carrying out various cultural activities for older people in the community, so as to promote the participation of older people and the accumulation of embodied cultural capital levels.

This study also has some limitations, which need to be improved in the future. Firstly, this study only focuses on embodied cultural capital, without considering the institutionalized cultural capital and objectified cultural capital in the relationship between the alienation and mental health. Secondly, this study mainly explored the mediating and moderating factors of alienation and embodied cultural capital. It is possible that there are other intermediate variables in the relationship between Internet use and mental health, and future research will further expand the study of mediating factors in the relationship. Thirdly, this study uses the cross-sectional data based on CGSS2017. Although the analysis and discussion are based on the existing research, we can not make a complete inference on the causality. Future research can explore the impact mechanism of Internet use on the mental health of the elderly within the framework of longitudinal development.
